# (2*E*)-1-(5-Bromo­thio­phen-2-yl)-3-(2,3,4-trimeth­oxy­phen­yl)prop-2-en-1-one

**DOI:** 10.1107/S1600536811052202

**Published:** 2011-12-10

**Authors:** K. Sunitha, H. C. Devarajegowda, Waleed Fadl Ali Al-eryani, Y. Rajendra Prasad, A. Uma Mahesh Kumar

**Affiliations:** a Institute of Pharmacy, GITAM University, Visakhapatnam 45, Andhrapradesh, India; b Department of Physics, Yuvaraja’s College (Constituent College), University of Mysore, Mysore 570 005, Karnataka, India; c Department of Pharmaceutical Chemistry, AU College of Pharmacy, Andhra University, Visakhapatnam, Andhrapradesh, India

## Abstract

In the title compound, C_16_H_15_BrO_4_S, the thio­phene ring is not coplanar with the benzene ring; the dihedral angle between the two planes is 11.08 (12)°. The crystal structure is characterized by C—H⋯O inter­actions. Weak intra­molecular C—H⋯O hydrogen bonds also occur.

## Related literature

For general background to chalcones, see: Chun *et al.* (2001 ▶); Horng *et al.* (2003 ▶); Lopez *et al.* (2001 ▶); Zubieta *et al.* (2001 ▶); Howard *et al.* (2004 ▶); Petrash (2004 ▶); Lu *et al.* (2010 ▶). Mei *et al.* (2003 ▶). For related structures, see: Liang *et al.* (2011) ▶; Alex *et al.* (1993 ▶); Li & Su (1993 ▶).
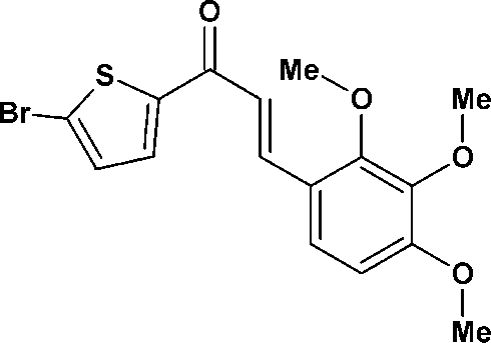


## Experimental

### 

#### Crystal data

C_16_H_15_BrO_4_S
*M* *_r_* = 383.25
Monoclinic, 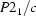

*a* = 8.114 (5) Å
*b* = 12.775 (5) Å
*c* = 15.404 (5) Å
β = 98.813 (5)°
*V* = 1577.9 (13) Å^3^
*Z* = 4
Mo *K*α radiation
μ = 2.75 mm^−1^
*T* = 293 K
0.22 × 0.15 × 0.12 mm

#### Data collection

Oxford Diffraction Xcalibur diffractometer
Absorption correction: multi-scan (*CrysAlis PRO RED*; Oxford Diffraction, 2010 ▶) *T*_min_ = 0.228, *T*_max_ = 1.000
16471 measured reflections
2784 independent reflections
2343 reflections with *I* > 2σ(*I*)
*R*_int_ = 0.042

#### Refinement

*R*[*F* ^2^ > 2σ(*F* ^2^)] = 0.033
*wR*(*F* ^2^) = 0.082
*S* = 1.06
2784 reflections
199 parameters
H-atom parameters constrained
Δρ_max_ = 0.33 e Å^−3^
Δρ_min_ = −0.59 e Å^−3^

### 

Data collection: *CrysAlis PRO CCD* (Oxford Diffraction, 2010 ▶); cell refinement: *CrysAlis PRO CCD*; data reduction: *CrysAlis PRO RED* (Oxford Diffraction, 2010 ▶); program(s) used to solve structure: *SHELXS97* (Sheldrick, 2008 ▶); program(s) used to refine structure: *SHELXL97* (Sheldrick, 2008 ▶); molecular graphics: *ORTEP-3* (Farrugia, 1997 ▶) and *CAMERON* (Watkin *et al.*, 1993 ▶); software used to prepare material for publication: *WinGX* (Farrugia, 1999 ▶).

## Supplementary Material

Crystal structure: contains datablock(s) I, global. DOI: 10.1107/S1600536811052202/zj2039sup1.cif

Structure factors: contains datablock(s) I. DOI: 10.1107/S1600536811052202/zj2039Isup2.hkl

Supplementary material file. DOI: 10.1107/S1600536811052202/zj2039Isup3.cml

Additional supplementary materials:  crystallographic information; 3D view; checkCIF report

## Figures and Tables

**Table 1:** Hydrogen-bond geometry (Å, °)

*D*—H⋯*A*	*D*—H	H⋯*A*	*D*⋯*A*	*D*—H⋯*A*
C8—H8*B*⋯O5	0.96	2.23	2.861 (4)	122
C9—H9*B*⋯O4	0.96	2.38	2.985 (4)	120
C21—H21⋯O6^i^	0.93	2.41	3.322 (4)	165

Symmetry code: (i) 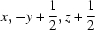.

**Fig. 1.**
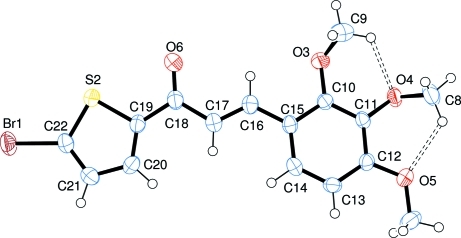
The molecular structure of the title compound. Displacement ellipsoids are drawn at the 50% probability level. The H atoms are shown as spheres of arbitrary radii. The dashed line indicates the intramolecular hydrogen bond.

**Fig. 2.**
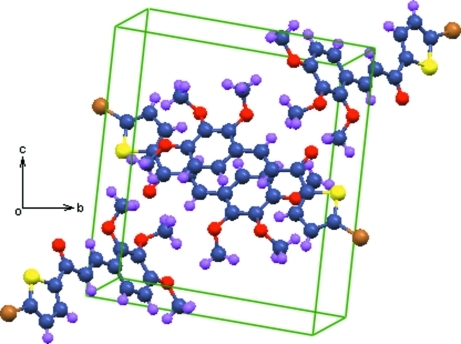
Packing of the molecules.

## supplementary crystallographic information

### Comment

Chalcones are alpha, beta unsaturated ketones, widely distributed in nature and are extensively studied for their biological activity (Chun *et al.*, 2001; Horng *et al.*, 2003; Lopez *et al.*, 2001; Mei *et al.*, 2003). Chalcones readily crystallize because of their intermolecular hydrogen bonding (Liang *et al.*, 2011; Alex *et al.*, 1993; Li *et al.*, 1993). The same property has been shown to be responsible for its biological activity (Zubieta *et al.*, 2001). However, halogen containing chalcones are of special interest in drug design process because of the raising importance of hlogen bond contribution in target recognition process (Howard *et al.*, 2004; Petrash, 2004; Lu *et al.*, 2010). Crystal structure conformation of small molecule has always been the choice for binding energy calculations in docking studies. In this paper we report the crystal structure of (2E)-1-(5-bromothiophen-2-yl)- 3-(2,3,4-trimethoxyphenyl)prop-2-en-1-one which is a part of insilico lead identification studies.

The asymmetric unit of (2E)-1-(5-bromothiophen-2-yl)-3-(2,3,4- trimethoxyphenyl)prop-2-en-1-one, C_16_H_15_Br_O_4_S_, contains just one molecule (Fig. 1). The five-membered thiophene ring (S2\C19\···C22) is not coplanar with the phenyl ring (C10\C11\···C15) system; the dihedral angle between the two planes is 11.08 (12)°. The crystal structure displays intermolecular C21—H21···O6 and weak intramolecular C8—H8B···O5 and C9—H9B···O4 hydrogen bonds (Table 1). The packing of molecules in the crystal structure is depicted in Fig. 2.

### Experimental

A mixture of 2-acetyl-5-BromoThiophene (0.01 mole) and 2,3,4- trimethoxybenzaldehyde (0.01 mole) were stirred in ethanol (30 ml) and then an aqueous solution of potassium hydroxide (40%,15 ml)was added to it. The mixture was kept over night at room temperature and then it was poured into crushed ice and acidified with dilute hydrochloric acid. The precipiteted chalcone was filtered and crystallized from ethanol.

### Refinement

All H atoms were positioned at calculated positions C—H = 0.93Å for aromatic H and C—H = 0.96Å for methyl H and refined using a riding model with *U*_iso_(H) = 1.2*U*_eq_(C)for aromatic and *U*_iso_(H) = 1.2*U*_eq_(C)for for methyl H.

### Crystal data

**Table:**

C_16_H_15_BrO_4_S	*F*(000) = 776
*M**_r_* = 383.25	*D*_x_ = 1.613 Mg m^−^^3^
Monoclinic, *P*2_1_/*c*	Melting point: 400 K
Hall symbol: -P 2ybc	Mo *K*α radiation, λ = 0.71073 Å
*a* = 8.114 (5) Å	Cell parameters from 2784 reflections
*b* = 12.775 (5) Å	θ = 2.5–25.0°
*c* = 15.404 (5) Å	µ = 2.75 mm^−^^1^
β = 98.813 (5)°	*T* = 293 K
*V* = 1577.9 (13) Å^3^	Prism, colourless
*Z* = 4	0.22 × 0.15 × 0.12 mm

### Data collection

**Table:**

Oxford Diffraction Xcalibur diffractometer	2784 independent reflections
Radiation source: fine-focus sealed tube	2343 reflections with *I* > 2σ(*I*)
graphite	*R*_int_ = 0.042
Detector resolution: 16.0839 pixels mm^-1^	θ_max_ = 25.0°, θ_min_ = 2.5°
ω scans	*h* = −9→9
Absorption correction: multi-scan (*CrysAlis PRO RED*; Oxford Diffraction, 2010)	*k* = −15→15
*T*_min_ = 0.228, *T*_max_ = 1.000	*l* = −18→18
16471 measured reflections	

### Refinement

**Table:**

Refinement on *F*^2^	Primary atom site location: structure-invariant direct methods
Least-squares matrix: full	Secondary atom site location: difference Fourier map
*R*[*F*^2^ > 2σ(*F*^2^)] = 0.033	Hydrogen site location: inferred from neighbouring sites
*wR*(*F*^2^) = 0.082	H-atom parameters constrained
*S* = 1.06	*w* = 1/[σ^2^(*F*_o_^2^) + (0.0349*P*)^2^ + 0.9331*P*] where *P* = (*F*_o_^2^ + 2*F*_c_^2^)/3
2784 reflections	(Δ/σ)_max_ = 0.007
199 parameters	Δρ_max_ = 0.33 e Å^−^^3^
0 restraints	Δρ_min_ = −0.59 e Å^−^^3^

### Special details

**Table:**

Experimental. *CrysAlis PRO*, Oxford Diffraction Ltd., Version 1.171.33.55 (release 05–01–2010 CrysAlis171. NET) Empirical absorption correction using spherical harmonics, implemented in SCALE3 ABSPACK scaling algorithm.
Geometry. All s.u.'s (except the s.u. in the dihedral angle between two l.s. planes) are estimated using the full covariance matrix. The cell s.u.'s are taken into account individually in the estimation of s.u.'s in distances, angles and torsion angles; correlations between s.u.'s in cell parameters are only used when they are defined by crystal symmetry. An approximate (isotropic) treatment of cell s.u.'s is used for estimating s.u.'s involving l.s. planes.
Refinement. Refinement of *F*^2^ against ALL reflections. The weighted *R*-factor *wR* and goodness of fit *S* are based on *F*^2^, conventional *R*-factors *R* are based on *F*, with *F* set to zero for negative *F*^2^. The threshold expression of *F*^2^ > 2σ(*F*^2^) is used only for calculating *R*-factors(gt) *etc*. and is not relevant to the choice of reflections for refinement. *R*-factors based on *F*^2^ are statistically about twice as large as those based on *F*, and *R*- factors based on ALL data will be even larger.

### Fractional atomic coordinates and isotropic or equivalent isotropic displacement parameters (Å^2^)

**Table:**

	*x*	*y*	*z*	*U*_iso_*/*U*_eq_	
Br1	−0.24507 (4)	0.00703 (2)	0.22076 (2)	0.05109 (14)	
S2	−0.11145 (9)	0.10763 (6)	0.06120 (5)	0.03737 (19)	
O3	0.3504 (3)	0.47747 (15)	−0.20529 (13)	0.0450 (5)	
O4	0.5493 (2)	0.65241 (14)	−0.19864 (12)	0.0378 (5)	
O5	0.5662 (2)	0.78703 (15)	−0.06147 (13)	0.0429 (5)	
O6	0.0059 (3)	0.22557 (18)	−0.08036 (14)	0.0561 (6)	
C7	0.5915 (4)	0.8532 (3)	0.0134 (2)	0.0527 (8)	
H7A	0.6611	0.9111	0.0026	0.079*	
H7B	0.6446	0.8143	0.0633	0.079*	
H7C	0.4859	0.8790	0.0250	0.079*	
C8	0.5199 (4)	0.7480 (2)	−0.2465 (2)	0.0566 (9)	
H8A	0.5915	0.7515	−0.2906	0.085*	
H8B	0.5428	0.8062	−0.2070	0.085*	
H8C	0.4056	0.7507	−0.2742	0.085*	
C9	0.2743 (4)	0.5175 (3)	−0.2882 (2)	0.0592 (10)	
H9A	0.2735	0.4642	−0.3322	0.089*	
H9B	0.3363	0.5769	−0.3036	0.089*	
H9C	0.1619	0.5383	−0.2847	0.089*	
C10	0.3613 (3)	0.5446 (2)	−0.13548 (17)	0.0301 (6)	
C11	0.4561 (3)	0.6350 (2)	−0.13228 (17)	0.0294 (6)	
C12	0.4695 (3)	0.6998 (2)	−0.05918 (17)	0.0305 (6)	
C13	0.3899 (3)	0.6724 (2)	0.01106 (17)	0.0351 (6)	
H13	0.3962	0.7162	0.0597	0.042*	
C14	0.3017 (3)	0.5805 (2)	0.00854 (18)	0.0340 (6)	
H14	0.2531	0.5616	0.0571	0.041*	
C15	0.2827 (3)	0.5150 (2)	−0.06390 (18)	0.0305 (6)	
C16	0.1893 (3)	0.4176 (2)	−0.06729 (19)	0.0358 (6)	
H16	0.1706	0.3834	−0.1212	0.043*	
C17	0.1279 (3)	0.3722 (2)	−0.00165 (19)	0.0387 (7)	
H17	0.1416	0.4057	0.0526	0.046*	
C18	0.0399 (3)	0.2720 (2)	−0.01058 (19)	0.0368 (7)	
C19	−0.0079 (3)	0.2265 (2)	0.06962 (18)	0.0331 (6)	
C20	0.0083 (3)	0.2636 (2)	0.15283 (19)	0.0401 (7)	
H20	0.0614	0.3264	0.1700	0.048*	
C21	−0.0624 (4)	0.1988 (2)	0.21091 (19)	0.0419 (7)	
H21	−0.0613	0.2134	0.2702	0.050*	
C22	−0.1317 (3)	0.1132 (2)	0.16997 (18)	0.0363 (7)	

### Atomic displacement parameters (Å^2^)

**Table:**

	*U*^11^	*U*^22^	*U*^33^	*U*^12^	*U*^13^	*U*^23^
Br1	0.0619 (2)	0.0412 (2)	0.0540 (2)	−0.00626 (15)	0.02110 (16)	0.01379 (15)
S2	0.0458 (4)	0.0314 (4)	0.0354 (4)	−0.0079 (3)	0.0077 (3)	0.0008 (3)
O3	0.0683 (14)	0.0315 (11)	0.0382 (12)	−0.0042 (10)	0.0176 (10)	−0.0070 (9)
O4	0.0486 (11)	0.0292 (11)	0.0407 (11)	−0.0010 (9)	0.0227 (9)	0.0033 (9)
O5	0.0549 (12)	0.0330 (12)	0.0421 (12)	−0.0122 (9)	0.0114 (9)	−0.0037 (9)
O6	0.0809 (16)	0.0551 (15)	0.0335 (12)	−0.0242 (12)	0.0124 (11)	0.0002 (11)
C7	0.059 (2)	0.0403 (19)	0.060 (2)	−0.0117 (16)	0.0121 (16)	−0.0162 (16)
C8	0.087 (3)	0.043 (2)	0.0442 (19)	−0.0002 (18)	0.0262 (18)	0.0127 (16)
C9	0.065 (2)	0.065 (2)	0.044 (2)	−0.0103 (18)	0.0000 (17)	−0.0121 (17)
C10	0.0364 (14)	0.0231 (14)	0.0320 (15)	0.0040 (11)	0.0093 (11)	0.0001 (11)
C11	0.0346 (14)	0.0260 (14)	0.0292 (14)	0.0040 (11)	0.0103 (11)	0.0053 (11)
C12	0.0336 (14)	0.0247 (14)	0.0333 (15)	0.0009 (11)	0.0058 (11)	0.0031 (12)
C13	0.0430 (16)	0.0350 (16)	0.0272 (14)	0.0009 (13)	0.0056 (12)	−0.0046 (12)
C14	0.0360 (15)	0.0367 (16)	0.0310 (15)	0.0009 (12)	0.0109 (11)	0.0057 (12)
C15	0.0296 (14)	0.0287 (15)	0.0340 (15)	0.0026 (11)	0.0073 (11)	0.0053 (12)
C16	0.0370 (15)	0.0327 (16)	0.0377 (16)	−0.0007 (12)	0.0061 (12)	0.0048 (13)
C17	0.0424 (16)	0.0383 (17)	0.0364 (16)	−0.0056 (13)	0.0093 (13)	0.0036 (13)
C18	0.0367 (15)	0.0373 (17)	0.0369 (17)	−0.0042 (12)	0.0070 (12)	0.0062 (13)
C19	0.0331 (14)	0.0303 (15)	0.0355 (16)	−0.0037 (11)	0.0036 (11)	0.0041 (12)
C20	0.0444 (16)	0.0334 (16)	0.0419 (18)	−0.0105 (13)	0.0043 (13)	−0.0023 (13)
C21	0.0514 (18)	0.0434 (18)	0.0319 (16)	−0.0024 (14)	0.0094 (13)	0.0016 (14)
C22	0.0383 (15)	0.0337 (16)	0.0386 (16)	0.0015 (12)	0.0113 (12)	0.0071 (13)

### Geometric parameters (Å, °)

**Table:**

Br1—C22	1.876 (3)	C10—C11	1.384 (4)
S2—C22	1.710 (3)	C10—C15	1.407 (4)
S2—C19	1.730 (3)	C11—C12	1.388 (4)
O3—C10	1.368 (3)	C12—C13	1.387 (4)
O3—C9	1.426 (4)	C13—C14	1.372 (4)
O4—C11	1.379 (3)	C13—H13	0.9300
O4—C8	1.428 (3)	C14—C15	1.384 (4)
O5—C12	1.366 (3)	C14—H14	0.9300
O5—C7	1.419 (3)	C15—C16	1.454 (4)
O6—C18	1.222 (3)	C16—C17	1.327 (4)
C7—H7A	0.9600	C16—H16	0.9300
C7—H7B	0.9600	C17—C18	1.462 (4)
C7—H7C	0.9600	C17—H17	0.9300
C8—H8A	0.9600	C18—C19	1.470 (4)
C8—H8B	0.9600	C19—C20	1.354 (4)
C8—H8C	0.9600	C20—C21	1.404 (4)
C9—H9A	0.9600	C20—H20	0.9300
C9—H9B	0.9600	C21—C22	1.342 (4)
C9—H9C	0.9600	C21—H21	0.9300
			
C22—S2—C19	90.49 (13)	C14—C13—C12	119.8 (3)
C10—O3—C9	116.5 (2)	C14—C13—H13	120.1
C11—O4—C8	117.0 (2)	C12—C13—H13	120.1
C12—O5—C7	118.5 (2)	C13—C14—C15	122.2 (2)
O5—C7—H7A	109.5	C13—C14—H14	118.9
O5—C7—H7B	109.5	C15—C14—H14	118.9
H7A—C7—H7B	109.5	C14—C15—C10	117.5 (2)
O5—C7—H7C	109.5	C14—C15—C16	122.6 (2)
H7A—C7—H7C	109.5	C10—C15—C16	119.9 (2)
H7B—C7—H7C	109.5	C17—C16—C15	126.9 (3)
O4—C8—H8A	109.5	C17—C16—H16	116.5
O4—C8—H8B	109.5	C15—C16—H16	116.5
H8A—C8—H8B	109.5	C16—C17—C18	123.1 (3)
O4—C8—H8C	109.5	C16—C17—H17	118.5
H8A—C8—H8C	109.5	C18—C17—H17	118.5
H8B—C8—H8C	109.5	O6—C18—C17	123.3 (3)
O3—C9—H9A	109.5	O6—C18—C19	119.6 (3)
O3—C9—H9B	109.5	C17—C18—C19	117.1 (3)
H9A—C9—H9B	109.5	C20—C19—C18	131.0 (3)
O3—C9—H9C	109.5	C20—C19—S2	110.7 (2)
H9A—C9—H9C	109.5	C18—C19—S2	118.2 (2)
H9B—C9—H9C	109.5	C19—C20—C21	114.0 (3)
O3—C10—C11	121.3 (2)	C19—C20—H20	123.0
O3—C10—C15	117.7 (2)	C21—C20—H20	123.0
C11—C10—C15	120.8 (2)	C22—C21—C20	111.3 (3)
O4—C11—C10	118.2 (2)	C22—C21—H21	124.3
O4—C11—C12	121.4 (2)	C20—C21—H21	124.3
C10—C11—C12	120.0 (2)	C21—C22—S2	113.4 (2)
O5—C12—C13	124.3 (2)	C21—C22—Br1	126.2 (2)
O5—C12—C11	116.1 (2)	S2—C22—Br1	120.36 (16)
C13—C12—C11	119.6 (2)		
			
C9—O3—C10—C11	−63.9 (3)	O3—C10—C15—C16	−2.1 (4)
C9—O3—C10—C15	120.7 (3)	C11—C10—C15—C16	−177.5 (2)
C8—O4—C11—C10	123.4 (3)	C14—C15—C16—C17	−8.3 (4)
C8—O4—C11—C12	−63.4 (3)	C10—C15—C16—C17	170.2 (3)
O3—C10—C11—O4	−4.4 (4)	C15—C16—C17—C18	−178.0 (3)
C15—C10—C11—O4	170.9 (2)	C16—C17—C18—O6	−5.1 (5)
O3—C10—C11—C12	−177.7 (2)	C16—C17—C18—C19	174.6 (3)
C15—C10—C11—C12	−2.5 (4)	O6—C18—C19—C20	−176.7 (3)
C7—O5—C12—C13	1.7 (4)	C17—C18—C19—C20	3.6 (4)
C7—O5—C12—C11	−176.7 (2)	O6—C18—C19—S2	−0.1 (4)
O4—C11—C12—O5	6.6 (4)	C17—C18—C19—S2	−179.73 (19)
C10—C11—C12—O5	179.7 (2)	C22—S2—C19—C20	0.8 (2)
O4—C11—C12—C13	−172.0 (2)	C22—S2—C19—C18	−176.6 (2)
C10—C11—C12—C13	1.2 (4)	C18—C19—C20—C21	176.4 (3)
O5—C12—C13—C14	−177.0 (2)	S2—C19—C20—C21	−0.5 (3)
C11—C12—C13—C14	1.4 (4)	C19—C20—C21—C22	−0.2 (4)
C12—C13—C14—C15	−2.8 (4)	C20—C21—C22—S2	0.8 (3)
C13—C14—C15—C10	1.5 (4)	C20—C21—C22—Br1	−178.3 (2)
C13—C14—C15—C16	−179.9 (2)	C19—S2—C22—C21	−0.9 (2)
O3—C10—C15—C14	176.5 (2)	C19—S2—C22—Br1	178.24 (17)
C11—C10—C15—C14	1.1 (4)		

### Hydrogen-bond geometry (Å, °)

**Table:**

*D*—H···*A*	*D*—H	H···*A*	*D*···*A*	*D*—H···*A*
C8—H8B···O5	0.96	2.23	2.861 (4)	122.
C9—H9B···O4	0.96	2.38	2.985 (4)	120.
C21—H21···O6^i^	0.93	2.41	3.322 (4)	165.

Symmetry codes: (i) *x*, −*y*+1/2, *z*+1/2.
